# Effects of Oculomotor Scanning on Agility Performance: Gender and a Type of Team Sport Comparison

**DOI:** 10.5114/jhk/196002

**Published:** 2025-07-21

**Authors:** Marek Popowczak, Teresa Zwierko

**Affiliations:** 1 Faculty of Physical Education and Sport, Wroclaw University of Health and Sport Sciences, Wroclaw, Poland.; 2 Institute of Physical Culture Sciences, Laboratory of Kinesiology, Functional and Structural Human Research Centre, University of Szczecin, Szczecin, Poland.

**Keywords:** visual search, team sport, performance, agility

## Abstract

There is limited evidence of recording eye movements in real-time while performing specific motor tasks in team games in response to environmental stimuli. This study aimed to examine how elite basketball and volleyball athletes adopted strategies during agility tasks, based on the type of team sports and gender. A total of 60 skilled players from both sports (30 males and 30 females), aged 16–18 years, participated in this study. Agility variables were assessed using the “Five-Time Shuttle Run to Gates” test, based on the “stop and go” protocol. To determine variables of fixation and saccade eye movements during the agility task, a mobile eye-tracking system was employed. MANOVA showed statistically significant main effects of gender and the type of team sport on agility (for both factors, p < 0.001, η^2^_part_ > 0.14) and on the number of saccades (η^2^_part_ = 0.16, p = 0.002; η^2^_part_ = 0.10, p = 0.018). Additionally, gender affected the number of fixations (η^2^_part_ = 0.08, p = 0.037). Regression analysis was used to explore the correlation between oculomotor variables and agility, indicating that for female basketball players, the number of saccades (ß = 1.04, B = 0.10) and the average velocity of saccades (ß = 0.79, B = 0.02) were the primary factors explaining agility variability. However, for male basketball players, agility test outcomes were largely determined by the duration of saccadic movements (ß = 0.79, B = 0.02). The findings suggest that the oculomotor scanning strategy in agility tasks significantly impacts athletic performance, and is influenced by the type of team sports and gender.

## Introduction

Given the high dynamics of playing offense and defense in team games, the use of effective visual activity strategies makes it possible to quickly search for meaningful signals from the environment. In dynamic and unpredictable match situations, there is a strong interaction between motor and perceptual processes, as athletes’ motor actions are often initiated by the perception, processing, and integration of environmental signals. These signals have a bearing on the movements of the player who constantly adapts their movements to complex temporal and spatial stimuli during the game (Zwierko et al., 2023). Therefore, it is important to determine oculomotor efficiency during team game-specific change of direction maneuvers (Popowczak et al., 2021; Young et al., 2021).

Oculomotor efficiency in sports refers to the effectiveness of controlling eye movements, which is essential for athletes performing tasks such as tracking moving objects, rapidly shifting focus, and maintaining stable vision during quick movements. This efficiency is directly tied to the athlete’s ability to execute tasks requiring precise timing and coordination, such as intercepting a ball or responding to fast-paced game scenarios. It is characterized by the speed and accuracy of eye movements, particularly during saccadic tasks (Ceyte et al., 2017; Jedziniak et al., 2019). Saccadic eye movements play a vital role in directing high-acuity foveal vision to important regions within the visual field, serving as the primary mechanism for visual search. These rapid shifts in fixation allow athletes to quickly acquire visual information by directing their gaze toward objects of interest (Marques et al., 2023; Zhao et al., 2012). In the context of sports, developing strong perception-movement correlations in specific agility tasks is crucial for optimal performance (Spiteri et al., 2018). This ability to swiftly adapt and respond to environmental constraints through precise oculomotor control is fundamental for effective performance in dynamic sports situations (Gegenfurtner et al., 2011; Hnidková et al., 2024; Kredel et al., 2017; Zwierko et al., 2018, 2024).

Agility is a skill that integrates both motor and perceptual decision-making components, making it a defining characteristic of team sports. To fully understand and enhance agility, it is essential to analyze it holistically, considering the interplay between physical movements and perceptual processing (Young et al., 2021). Although motor factors determining agility are often described in the literature as variables of change of direction speed (CODS; Scanlan et al., 2021), little is currently known about the key factors that characterize the perceptual component of this ability (Born et al., 2017; Zwierko et al., 2023). Perceptual processing is the process that involves combining information collected from sensory systems and using it to guide behavior, such as movement (Green and Heekeren, 2009). One of the key factors in perceptual processing is oculomotor control. During fixation, the athlete’s vision stabilizes on the fovea, which allows them to obtain important information that permits them to optimally direct their behavior. In turn, the athlete’s rapid scanning of the visual field is facilitated by saccades or rapid eye movements between one fixation and the next (Piras et al., 2014; Zwierko et al., 2019).

Many studies have confirmed that sports require rapid changes in fixation in the visual field, supporting the fact that effective saccadic eye movements are required (Ceyte et al., 2017; Jedziniak et al., 2019; Piras et al., 2010; Zwierko et al., 2019). Therefore, it is important to determine oculomotor performance in specific tests for team sports (e.g., basketball and volleyball), determining agility levels based on “stop and go” movement patterns. These schemes are based on the most commonly manifested ways players move during matches, i.e., repeated multi-directional acceleration and deceleration combined with a change in the direction of movement. So far, the problem of determining oculomotor control in the context of these agility tasks has not been addressed. In addition, laboratory procedures used to measure oculomotor abilities have been limited due to the lack of congruence between the testing tasks and the requirements of realistic environments (i.e., ecological validity; Kullmann et al., 2021; Popowczak et al., 2016).

Considering the necessity for further exploration of this issue under more sport-specific conditions, this study attempted to examine whether oculomotor function influences agility performance. Based on the authors’ knowledge, eye movements have not been recorded to light signals as stimuli from the environment using a mobile eye tracker device in agility tasks. This type of stimuli allows for a more accurate analysis of eye movements in visual search during players’ movements similar to those recorded during the game. In addition, most studies using eye-tracking systems in team sports players have been limited to tests in static positions: sitting or standing (Jin et al., 2023; Krzepota et al., 2016). There is a lack of research on how these functions influence agility in dynamic sports settings. On the other hand, the development of portable eye trackers allows a more accurate assessment of eye tracking (with a sampling rate of 100 Hz) during movement-based behaviors (Kredel et al., 2017).

Therefore, the primary objective of this study was to investigate how elite basketball and volleyball players utilized oculomotor scanning strategies during agility tasks and to determine whether these strategies differed based on the sports discipline and gender. Moreover, due to the height of the ball’s trajectory during in-game action, representatives of different sports would differ in their oculomotor functions with a static light stimulus presented at one height from the ground; therefore, we hypothesized that elite athletes would show distinct oculomotor strategies due to sport-specific training and expected to observe gender differences in saccadic speed (Bargary et al., 2017). Additionally, we explored the relationship between gaze variables and agility test performance. We hypothesized that, due to adaptations stemming from long-term training under the specific conditions typical of their sports discipline, athletes would exhibit distinct oculomotor strategies as a manifestation of experience-dependent plasticity (Stone et al., 2019). Specifically, we addressed three key questions regarding the correlation between gaze strategy variables and the execution time of agility tasks for the studied groups: (1) Did an increase in the number and duration of fixations lead to longer completion times in the agility test? (2) Did faster saccadic eye movements contribute to shorter trial times? (3) How did the eye mobility index influence the time required to complete the agility test?

## Methods

### Participants

The study group consisted of 60 skilled athletes, including 15 female basketball players (age: 21.7 ± 1.6 years; body height: 174.5 ± 7.8 cm; body mass: 68.0 ± 9.1 kg), 15 male basketball players (age: 22.3 ± 1.7 years; body height: 190.6 ± 6.6 cm; body mass: 89.0 ± 12.5 kg), 15 female volleyball players (age: 21.0 ± 1.2 years; body height: 172.9 ± 6.4 cm; body mass: 67.5 ± 7.4 kg), and 15 male volleyball players (age: 21.1 ± 1.8 years; body height: 187.1 ± 4.5 cm; body mass: 79.7 ± 9.2 kg). The study group included athletes who played in an academic league and had significant sports achievements in Poland (participants in the final phase of the University Polish Championships during the 2021/2022 sports season). Inclusion criteria included players participating in training at least four times per week before the start of the study. In addition, participants with normal or corrected-to-normal vision (by wearing contact lenses) and able to perform the test without the aid of glasses were included. The pilot study’s sample size of 60 participants was determined based on Viechtbauer et al. (2015). Before the study, athletes were excluded if they reported any musculoskeletal injuries, pain syndromes or other conditions that would be exacerbated by their participation in the measurement survey within the past year. In addition, eyeglass wearers were excluded from the study because eyeglasses affect the quality of eye-tracking registration.

This study was approved by the Research Bioethics Committee of the Faculty Senate of the Wroclaw University of Health and Sport Sciences (approval code: 23/2021; approval date: 31 December 2021) and was conducted following the ethical principles for medical research involving human subjects contained in the Declaration of Helsinki by the World Medical Association. This study also met the “Ethical standards in sport and exercise science research” (Harriss et al., 2022). All participants were asked to provide written informed consent before the study, and the purpose and characteristics of the research were explained.

### Measures

All measurements were taken in the sports hall where participants usually played and trained. Therefore, under such conditions, each participant performed the test at the maximum level. Participants were familiar with all measurements. Anthropometric measurements and assessment of motor skills were carried out following the procedures of the Central Laboratory of the Wroclaw University of Health and Sport Sciences (ISO PN-EN ISO 9001:2015). On the other hand, measurements of oculomotor performance were carried out by an employee from another institution specialized in conducting such tests.

### Design and Procedures

All the measurements were completed in one day. Athletes’ body height was measured with a GPM 101 anthropometer (DKSH, Zurich, Switzerland) with accuracy of 1 mm. Participants’ body mass was measured when they were modestly dressed and without shoes, using the InBody 230 system (Tanita Corp., Tokyo, Japan). Before measuring agility and oculomotor performance, participants completed a standard 15-min warm-up procedure without eye trackers.

**Figure 1:**
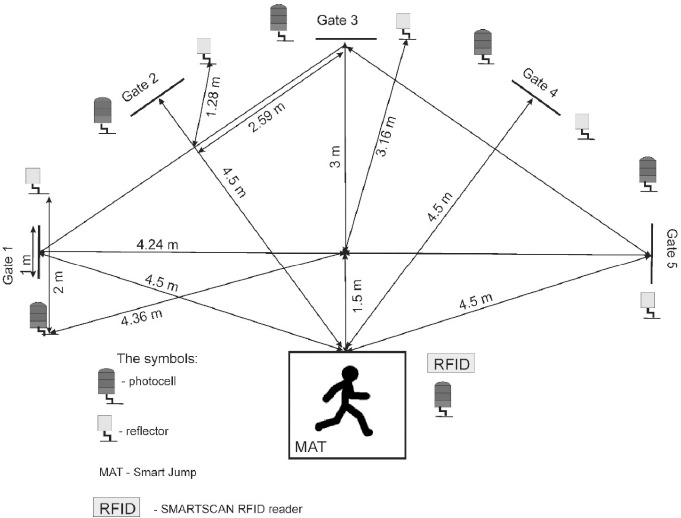
“Five-Time Shuttle Run to Gates” test.

Agility variables were determined by the “Five-Time Shuttle Run to Gates” test based on a “stop and go” scheme (Popowczak et al., 2020). A Fusion Smart Speed system (Fusion Sport, Coopers Plains, QLD, Australia) was used during the test. The system consisted of photocells integrated with a Smart Jump mat’s photocell and an application of the Smartspeed Timing Gate System on an iPad (Figure 1). The total test time was recorded to the nearest 0.01 s. The test data (participant code) was stored in the Smartspeed Timing Gate System application (v 1.0.1) and downloaded to a Dell laptop for subsequent analysis. All data were then exported to a Microsoft Excel spreadsheet (version 2017).

The “Five-Time Shuttle Run to Gates” test measures the time of repeated “stop and go” directional changes responding to a light signal, following the procedures proposed by Popowczak et al. (2016). In the test, the participant had to run the distance from the mat to the gate’s line (1 m) and return to the mat five times. Each gate consisted of a photocell and a reflector set 2 m apart. The mat had integrated photocells. When the researcher said “start”, the participant jumped on the mat with both feet. After touching the mat with both feet, the participant received a light signal randomly indicating the goal of the run (the gate). The trial time was measured from the moment both feet touched the mat. The participant ran to the goal, crossed the line (1 m long) with both feet, and immediately ran back to the mat (Smart Jump). After contact with the mat with both feet, another randomly selected light was lit. Participants repeated this cycle five times until, after touching the mat with both feet, no light signal in the gates illuminated, and the researcher said “stop”, ending the trial. The agility test was repeated four times. Each participant rested for 3 min between repetitions in each test. The best results (total time) of the run in the tests were used in the analysis. In all tests, participants covered the same distances. The reliability analysis of the “Five-Time Shuttle Run” test results indicated statistically significant reliability for agility (Cronbach’s alpha: 0.932).

To determine oculomotor performance during the agility task, a mobile eye tracking system was used—Eye-Trackers (head-mounted infra-red Tobii Pro Glasses 2, Tobii Technology Inc. VA, USA), which allowed the measurement of fixation variables and saccadic eye movements. The Tobii system extracted the participant’s gaze coordinates by recording the participants’ pupils at a sampling rate of 100 Hz using infrared illumination. In addition, on the front of the glasses’ frames there was a camera that recorded video in Full HD resolution (25 fps, H.264) at a 50- or 100-Hz refresh rate and a 90-degrees field of the view. The data recorded by the glasses were saved using Tobii Glasses Controller Software (v. 1.114).

The eye-tracking device was calibrated before each performed test by the test subject using the manufacturer’s single-point calibration method. To calibrate the Tobii Pro eye-movement system, the participant focused on the center of the calibration card, held in front of them at a distance of 1 m for 5–15 s. After successful calibration was verified, participants were free to look on while waiting for the agility task to begin. In addition, participants were instructed to perform the test as quickly as possible following its procedures. After the trial was completed, eye-tracking data were stored in the Tobii system. The raw eye tracking data (gaze coordinates: x, y) from the fastest agility trial (recorded between the researcher’s words: “start” and “stop”) were used in further analyses. The data were extracted from the Tobii Pro Glasses 2 and processed using Pro Lab Software (v .1.181).

Recorded fixation was defined as a stable eye position maintained for at least 60 ms. Saccades were defined as eye movement between two fixation points with amplitudes greater than 1 degree. In addition, fixation fusion was performed according to the procedure described by Hooge et al. (2022). The following gaze variables were analyzed: the number of fixations [n], fixation duration [ms], the number of saccades [n], saccade duration [ms], average saccade velocity [^o^/s], and the ocular mobility index. The ocular mobility index (%) was calculated using the formula 100 × (saccade duration/[fixation duration + saccade duration]), according to Poiroux et al. (2015).

### Statistical Analysis

The Shapiro-Wilk test was used to evaluate the normality of the distribution of the continuous variables, while the Levene’s test was used to assess homoscedasticity. The variables showed a normal distribution and equality of the variances. All variables were described as means, SDs, and with 95% confidence intervals (95% CI).

Between-group (male and female basketball players, male and female volleyball players) differences in the measurement of agility and gaze variables were computed using MANOVA. The main effects (gender and the type of team sport) were examined, as well as the interaction term (gender*type of team sport). The effect size was assessed using partial eta squared (η^2^_part_) and interpreted as η^2^_part_ = 0.01, small effect; η^2^_part_ = 0.06, medium effect; and η^2^_part_ = 0.14 indicating a large effect. When significant differences were observed, ANOVA with detailed post hoc test comparisons (Bonferroni Post Hoc tests) was used. Statistical significance was set at an α level of 0.05.

The next step in the analysis was to study the correlation (dependence) between oculomotor variables and agility, and to this end, linear regression was used. The quality of the regression model was evaluated with adjusted R^2^ and linear coefficients (B), and standardized coefficients (β) were calculated. In all calculations, Statistica software version 13.0 (TIBCO Software Inc., Palo Alto, CA, USA) was employed.

## Results

The results (mean ± SD) of the oculomotor and agility functional performance tests are provided for gender and the type of team sports discipline groups in Table 1.

**Table 1:** Participant characteristics by gender and sport categories. Means, standard deviations, and 95% CI are presented.

Variables	Sports	Females (n = 30)		Males (n = 30)	
		Basketball (n = 15)	Volleyball (n = 15)	Basketball (n = 15)	Volleyball (n = 15)
Agility [s]	Mean ± SD	19.28 ± 1.21	20.00 ± 1.03	17.60 ± 0.93	18.75 ± 0.67
	(95% CI)	(18.62–19.95)	(19.43–20.57)	(17.08–18.11)	(18.38–19.12)
Number of fixations [n]	Mean ± SD	22.20 ± 8.17	26.80 ± 10.09	19.60 ± 5.95	20.93 ± 5.64
	(95% CI)	(17.68–26.72)	(21.21–32.39)	(16.31–22.89)	(17.81–24.06)
Fixation duration [ms]	Mean ± SD	90.72 ± 15.48	91.07 ± 16.47	99.02 ± 23.57	94.95 ± 15.00
	(95% CI)	(82.15–99.30)	(81.95–100.20)	(85.96–112.07)	(86.64–103.26)
Number of saccades [n]	Mean ± SD	55.27 ± 11.50	65.27 ± 8.61	51.93 ± 5.12	53.27 ± 9.52
	(95% CI)	(48.90–61.64)	(60.50–70.04)	(49.10–54.77)	(47.99–58.54)
Saccade duration [ms]	Mean ± SD	260.63 ± 57.33	230.24 ± 44.34	252.92 ± 43.93	252.36 ± 62.21
	(95% CI)	(228.88–292.38)	(205.69–254.80)	(228.59–277.24)	(217.90–286.81)
Saccade velocity average [^o^/s]	Mean ± SD (95% CI)	293.70 ± 47.64	265.25 ± 46.03	297.39 ± 41.80	310.95 ± 60.73
		(267.32–320.08)	(239.76–290.74)	(274.24–320.54)	(277.32–344.59)
Ocular mobility index [%]	Mean ± SD	87.05% ± 4.77%	85.44% ± 6.64%	87.06% ± 4.73%	86.87% ± 3.09%
	(95% CI)	(84.41%–89.69%)	(81.76%–89.11%)	(84.44%–89.68%)	(85.16%–88.58%)

Significant main effects of gender and the type of team sport for agility were found for both factors (*p* < 0.001, η^2^_part_ > 0.14, large effect). Similarly, a significant main effects of gender (large effect, *p* < 0.01) and type of team sport (medium effect, *p* < 0.05) were observed for the number of saccades (Table 2). The effect of gender was greater than the type of team sport. Besides the fact that gender affected fixation (medium effect, *p* < 0.05), there was no significant effect of the type of team sport. There were no significant effects of the independent variables on the other dependent visual variables (*p* > 0.05), as well as any of the interaction terms.

**Table 2:** Differences according to gender and the type of team sports of the analyzed variables (MANOVA statistical analysis).

Variable	Gender		Type of Team Sports		Gender*Type of Team Sports	
	*p-value*	η^2^_part_	*p-value*	η^2^_part_	*p-Value*	η^2^_part_
Agility [s]	<0.001	0.38	<0.001	0.20	0.388	0.01
Number of fixations [n]	0.037	0.08	0.140	0.04	0.413	0.01
Fixation duration [ms]	0.194	0.03	0.690	0.01	0.636	0.01
Number of saccades [n]	0.002	0.16	0.018	0.10	0.067	0.06
Saccade duration [ms]	0.598	0.01	0.259	0.02	0.277	0.02
Saccade velocity average [^o^/s]	0.058	0.06	0.563	0.01	0.106	0.05
Ocular mobility index [%]	0.575	0.01	0.485	0.01	0.583	0.01

Notes: η^2^_part_: partial eta square

Detailed comparisons between all four groups (Bonferroni Post Hoc) showed several differences. In the agility test, male basketball players achieved a significantly shorter time (*p* < 0.001) than female basketball players (by 1.68 s), female volleyball players (by 2.40 s), and male volleyball players (by 1.15 s). Volleyball players, on the other hand, were faster in the motor test than volleyball players by 1.25 s (*p* < 0.01).

Analysis of oculomotor control variables made it possible to note only statistically significant differences in the number of saccades between groups differentiated by gender and the type of team sport. Female volleyball players differed by having more saccades than female basketball players (by n = 10.00, *p* = 0.02), male basketball players (by n = 13.33, *p* < 0.001), and male volleyball players (by n = 12.00, *p* = 0.003). In addition, we noted that significant gender differences in the number of fixations were not confirmed by the post hoc analysis. However, it was observed that female basketball and volleyball players had fewer fixations during the agility test than male athletes.

Tests of the correlations between statistically significant oculomotor variables and agility were examined by regression. Analyses of the correlations between variables included a breakdown by gender and the type of team sport. It was observed that saccade variables in basketball players significantly altered agility performance. In contrast, fixation and the eye mobility index did not affect trial times.

The regression models built (for female and male basketball players) were statistically significant and well-fitted to the explanatory variables (adjusted R^2^ = 0.55. F = 6.74, *p* = 0.008; adjusted R^2^ = 0.45. F = 4.82 *p* =0.022), although the men’s model presented a slightly better fit. In female basketball players, variability in agility was mostly explained by the number of saccades (ß = 1.04, B = 0.10) and the average velocity of the saccades (ß = 0.79, B = 0.02). However, in male basketball players, the motor test scores were primarily explained by the duration of saccadic movements (ß = 0.79, B = 0.02). Higher values of these variables resulted in longer agility test times among basketball players. The regression models built for volleyball players were not statistically significant.

## Discussion

This was the first study to determine the function of tracking eye movements during acceleration and deceleration in agility tests. Additionally, the assessment of the visual strategy was conducted using a task based on a “stop and go” design, in which the subject’s movement pattern is similar to his or her activity on the court during the game. Our study indicates that the adopted oculomotor scanning strategy during agility tasks may affect athletes’ performance. This was confirmed in a group of male and female basketball players where a greater number and longer duration of saccades were associated with an increased time to complete reactive agility tasks. Consequently, it appears that the “saccadic” strategy is less effective in searching for signals within a 180-degree field of vision. Previous research (Gegenfurtner et al., 2011) suggests that experienced athletes initiate their saccades later, which results in shorter saccade duration in task-relevant areas, and they also exhibit greater saccadic amplitudes. Additionally, studies (Klatt and Smeeton, 2022; Klostermann et al., 2019) suggest that in scenarios where athletes need to monitor information from various locations, peripheral vision tends to be more effective than saccadic movements. This is particularly evident when athletes are involved in simultaneous tasks such as detecting changes in motion (Klostermann et al., 2019) Additionally, peripheral vision allows the assessment of the teammates’ and opponents’ position as the ball moves in play, leading to overall better decision-making (Klatt and Smeeton, 2022). Therefore, introducing tasks aimed at integrating peripheral and foveal signals at the same time while a player observes the court is a challenge for coaches preparing training programs (Krzepota et al., 2015).

In light of our findings, it is important to identify the optimal visual strategy in the context of agility movements that have practical implications for sports training. Three main gaze patterns that make use of both foveal and peripheral vision in sports settings have been recognized: maintaining fixation on a central location (foveal spot), using a visual pivot for the detection of peripheral events, and employing a gaze anchor to position the gaze in free space while monitoring the movement of objects with peripheral vision (Klostermann et al., 2019). It is important to note that differences between foveal and peripheral vision exist physiologically and perceptually. Nevertheless, despite these differences, processing peripheral and foveal vision is not independent but closely interconnected (Stewart et al., 2020). Considering this, developing perceptual-motor training programs that foster optimal visual strategies during agility tasks to enhance an athlete’s performance may be valuable.

Our research has shown that agility, as well as visual strategies, vary across different sports disciplines. Specifically, basketball players demonstrated better performance in reactive agility tasks compared to volleyball players, which was associated with a smaller number of saccades. Our results confirm earlier reports of the differences in performance in reactive agility tasks among representatives of various sports disciplines (Domaradzki et al., 2021; Zemková, 2016). To maintain the integrity of specific sports skills in a game environment, agility movements in team sports demand a significant integration of the athlete’s perceptual-cognitive and motor subsystems (Farrow and Abernethy, 2003). However, it has been indicated that sport-specific demands differentiate between perceptual-cognitive and motor components in agility tasks. Zemková (2016) found that karate-kumite competitors and hockey and soccer goalies had a higher agility index compared to karate competitors and field players in hockey and soccer. A higher agility index suggests a balanced use of perceptual-cognitive and motor components during performance, while lower values indicate greater reliance on speed abilities. Adaptation to sport-specific demands has been confirmed in earlier studies on oculomotor function. Zwierko et al. (2019) found significantly better saccadic variables in skilled soccer players compared to non-athletes, particularly they achieved higher values in the following saccadic characteristics: average acceleration, acceleration peak, deceleration peak, and average velocity. Similarly, Piras et al. (2010) pointed out that faster oculomotor dynamics in volleyball players were due to expert volleyball players extracting more task-relevant information from each fixation than less skilled athletes, and that players’ proficiency influenced the strategies used in processing visual information.

Our study results indicated gender differences stemming from both agility performance and oculomotor function. These results align with existing research, which shows that males typically have faster agility performances than females (Spiteri et al., 2014; Zwierko et al., 2023). The disparity in agility tasks between genders is largely ascribed to factors such as neuromuscular traits (Landry et al., 2009), lower limb strength (Spiteri et al., 2013), exit velocity, and ground reaction forces (Condello et al., 2016), along with possible variations in perceptual-cognitive processing. Specifically, Spiteri et al. (2014) observed that in team sports, male athletes exhibited significantly quicker agility movements, generating more force and impulse, and showing greater trunk and knee flexion angles than females, leading to notable gender differences in decision-making time and post-stride velocity.

Regarding oculomotor function, we observed that the main difference between groups pertained to the number of saccadic movements, where female volleyball players exceeded both male volleyball and basketball players as well as female basketball players. In contrast, Zwierko et al. (2023) found that male volleyball players, compared to female players, had significantly better saccade dynamics, although other perceptual-cognitive factors (such as selective attention, simple reaction time, complex reaction time, and sensory sensitivity) showed non-significant or small to moderate differences in relation to gender. Shaqiri et al. (2018) observed that differences between genders in visual and perceptual functions varied widely. In their study involving fifteen distinct visual tasks, it was found that males significantly outperformed females in areas such as simple reaction time, visual acuity, visual backward masking, detecting motion direction, interpreting biological motion, and perceiving the Ponzo illusion. Our findings call for additional studies to validate the existence of gender-related disparities in oculomotor control during agility tasks.

This study marks progress in our understanding of the oculomotor control processes during agility field testing, yet it is not without limitations that need to be addressed. Some studies have analyzed the impact of visuo-motor processing on agility variables. For example, Zemková (2016), Horníková et al. (2021), and Popowczak et al. (2020) noted that reaction time and decision-making time influenced agility outcomes. Our current research focused primarily on the impact of oculomotor function on agility performance, which does not explain all cognitive aspects (Krzepota et al., 2015). Secondly, saccades were treated as a unified entity, which limited the exploration of the influence of catch-up saccades on the adopted gaze strategies. Thirdly, our study participants presented an amateur performance level, therefore, generalizing conclusions to other populations, for example elite athletes, is not feasible. Future studies should continue among athletes of a higher sports level. Lastly, in assessing agility, we utilized a non-specific (light) stimulus. While the primary advantage of this type of stimulus is its ability to be consistently programmed to appear at the same time during each test, enhancing reproducibility (Paul et al., 2016), it has been suggested that agility tests using specific (human) stimuli might better reflect real-world scenarios, thereby offering greater ecological validity and relevance to actual sports performance (Morral-Yepes et al., 2022).

## Conclusions

The choice of oculomotor scanning strategies in agility tasks significantly influences athletic performance. The variance in performance is dependent on both the specific sports discipline and the athlete’s gender. This research sheds light on new approaches to employing oculomotor scanning strategies tailored to the unique demands of different sports environments.
